# The *Quest for Orthologs* benchmark service and consensus calls in 2020

**DOI:** 10.1093/nar/gkaa308

**Published:** 2020-05-06

**Authors:** Adrian M Altenhoff, Javier Garrayo-Ventas, Salvatore Cosentino, David Emms, Natasha M Glover, Ana Hernández-Plaza, Yannis Nevers, Vicky Sundesha, Damian Szklarczyk, José M Fernández, Laia Codó, the Quest for Orthologs Consortium, Josep Ll Gelpi, Jaime Huerta-Cepas, Wataru Iwasaki, Steven Kelly, Odile Lecompte, Matthieu Muffato, Maria J Martin, Salvador Capella-Gutierrez, Paul D Thomas, Erik Sonnhammer, Christophe Dessimoz

**Affiliations:** SIB Swiss Institute of Bioinformatics, Lausanne, Switzerland; ETH Zurich, Department of Computer Science, Zurich, Switzerland; Life Sciences Department, Barcelona Supercomputing Center (BSC), Barcelona, Spain; Department of Biological Sciences, Graduate School of Science, The University of Tokyo, Tokyo, Japan; Department of Plant Sciences, University of Oxford, South Parks Road, Oxford, UK; SIB Swiss Institute of Bioinformatics, Lausanne, Switzerland; Department of Computational Biology, University of Lausanne, Lausanne, Switzerland; Center for Integrative Genomics, University of Lausanne, Lausanne, Switzerland; Centro de Biotecnologia y Genomica de Plantas, Universidad Politécnica de Madrid (UPM) - Instituto Nacional de Investigación y Tecnología Agraria y Alimentaria (INIA), Campus de Montegancedo-UPM, 28223, Pozuelo de Alarcón, Madrid, Spain; SIB Swiss Institute of Bioinformatics, Lausanne, Switzerland; Department of Computational Biology, University of Lausanne, Lausanne, Switzerland; Center for Integrative Genomics, University of Lausanne, Lausanne, Switzerland; Department of Computer Science, ICube, UMR 7357, University of Strasbourg, CNRS, Fédération de Médecine Translationnelle de Strasbourg, Strasbourg, France; Life Sciences Department, Barcelona Supercomputing Center (BSC), Barcelona, Spain; SIB Swiss Institute of Bioinformatics, Lausanne, Switzerland; Institute of Molecular Life Sciences, University of Zurich, Winterthurerstrasse 190, Zurich, 8057, Switzerland; Life Sciences Department, Barcelona Supercomputing Center (BSC), Barcelona, Spain; Life Sciences Department, Barcelona Supercomputing Center (BSC), Barcelona, Spain; Life Sciences Department, Barcelona Supercomputing Center (BSC), Barcelona, Spain; Department of Biochemistry and Molecular Biomedicine. University of Barcelona. Barcelona, Spain; Centro de Biotecnologia y Genomica de Plantas, Universidad Politécnica de Madrid (UPM) - Instituto Nacional de Investigación y Tecnología Agraria y Alimentaria (INIA), Campus de Montegancedo-UPM, 28223, Pozuelo de Alarcón, Madrid, Spain; Department of Biological Sciences, Graduate School of Science, The University of Tokyo, Tokyo, Japan; Department of Plant Sciences, University of Oxford, South Parks Road, Oxford, UK; Department of Computer Science, ICube, UMR 7357, University of Strasbourg, CNRS, Fédération de Médecine Translationnelle de Strasbourg, Strasbourg, France; European Molecular Biology Laboratory, European Bioinformatics Institute, Wellcome Genome Campus, Hinxton, Cambridge, UK; European Molecular Biology Laboratory, European Bioinformatics Institute, Wellcome Genome Campus, Hinxton, Cambridge, UK; Life Sciences Department, Barcelona Supercomputing Center (BSC), Barcelona, Spain; Division of Bioinformatics, Department of Preventive Medicine, University of Southern California, Los Angeles, USA; Science for Life Laboratory, Department of Biochemistry and Biophysics, Stockholm University, Solna, Sweden; SIB Swiss Institute of Bioinformatics, Lausanne, Switzerland; Department of Computational Biology, University of Lausanne, Lausanne, Switzerland; Center for Integrative Genomics, University of Lausanne, Lausanne, Switzerland; Department of Genetics, Evolution & Environment, University College London, London, UK; Department of Computer Science, University College London, London, UK

## Abstract

The identification of orthologs—genes in different species which descended from the same gene in their last common ancestor—is a prerequisite for many analyses in comparative genomics and molecular evolution. Numerous algorithms and resources have been conceived to address this problem, but benchmarking and interpreting them is fraught with difficulties (need to compare them on a common input dataset, absence of ground truth, computational cost of calling orthologs). To address this, the *Quest for Orthologs* consortium maintains a reference set of proteomes and provides a web server for continuous orthology benchmarking (http://orthology.benchmarkservice.org). Furthermore, consensus ortholog calls derived from public benchmark submissions are provided on the *Alliance of Genome Resources* website, the joint portal of NIH-funded model organism databases.

## INTRODUCTION

The identification of orthologs—pairs of genes in different species which have evolved from a common gene in the last ancestor of the species (1)—is essential for a wide range of analyses in phylogenetics and comparative genomics (reviewed in 2). However, because ortholog inference requires reconstructing evolutionary events which have taken place in single genes potentially hundreds of millions of years in the past, inference can be a challenging task (reviewed in 3).

For over 10 years, the *Quest for Orthologs* consortium has brought together orthology method developers, orthology and model organism database providers, and orthology users to improve inference, provision and interpretation of ortholog data (4–8). Among the main achievements of the consortium are agreements on standard data file format for orthology (9), an orthology ontology (10,11), curation of a reference set of proteomes (5), curation of a reference species tree (12) and community-led benchmarking (13).

However, the available genomes and methods change over time, requiring both reference proteomes and benchmarking to be continuously updated and re-interpreted. Furthermore, since benchmarking requires gathering and comparing ortholog calls obtained from various methods, there is an opportunity to provide the community with consensus calls.

Here, we provide updates of the *Quest for Orthologs* Reference Proteomes and benchmark service, and introduce a new ortholog consensus service, which is integrated to the website of the *Alliance for Genome Resources*—the joint portal of NIH-funded model organism databases (14).

## REFERENCE PROTEOMES

Orthology benchmarking needs a consensus dataset of proteomes and common file formats to be used by the different orthology prediction methods for standardized benchmarks and result interpretation. Since 2011, the Quest for Orthologs (QfO) makes available a Reference Proteomes dataset, providing a representative protein for each gene in the genome of selected species. These datasets have been generated annually from the UniProt Knowledgebase (UniProtKB) (15) using an automatic gene-centric pipeline which identifies all protein isoforms for a gene and selects the canonical protein sequence as representative of the set. The canonical sequences are typically the longest isoform which best describes the sequence annotations e.g. domains, isoforms, polymorphisms, post-translational modifications (https://www.uniprot.org/help/canonical_and_isoforms). The QfO community has been working with UniProt over the years to identify species for the reference data set, and now comprises well-annotated model organisms, organisms of interest for biomedical research and species broadly covering the tree of life for phylogenetic interpretation. The QfO Reference Proteomes are a manually compiled subset of the UniProt Reference Proteomes and their generation is now an integral part of the UniProt release pipelines.

The QfO Reference Proteomes dataset is updated every year and the latest version (2019) comprises 78 species (48 Eukaryotes, 23 Bacteria and 7 Archaea) that are based on the UniProtKB 2019_04 release of 8 May 2019. In total, this represents 991,290 protein sequences. The set of included species has remained stable over the years, but the protein sequences fluctuate more rapidly due to improvements in UniProtKB annotations. The number of species included is a compromise between keeping the dataset manageable for the benchmarking efforts, representing the available protein sequence space, and including the model species of highest interest to the research community. For the species included, a phylogenetic tree reflecting the consensus in the literature is maintained alongside the Reference Proteomes (12). The Quest for Orthologs Reference Proteomes are available for download in different formats: the protein sequences as FASTA and SeqXML files, CDS sequences for most proteins as FASTA files, and, for an increasing number of species, the genomic locus coordinates are available in XML format.

The benchmark service aims to follow the yearly release cycle of the Reference Proteome datasets, with a lag of a few months. Users are encouraged to always benchmark against the latest available dataset. In this publication, we will discuss the results on the 2018 Reference Proteome dataset.

## BENCHMARK SERVICE

**Figure 1.**
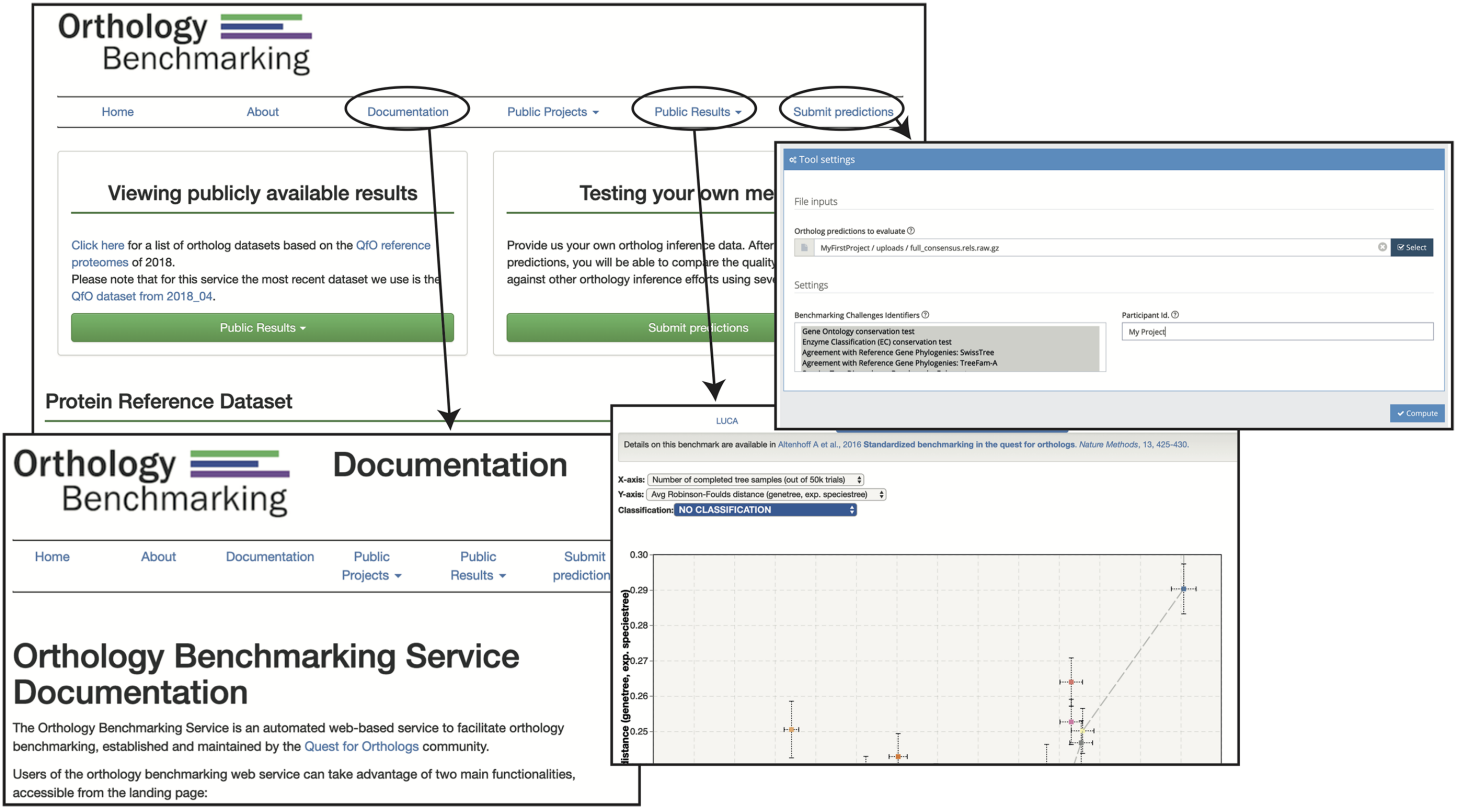
Overview of the https://orthology.benchmarkservice.org website. Benchmarks are now computed using the OpenEBench cloud-based platform from ELIXIR.

As introduced in (13), the Quest for Orthologs benchmark service takes ortholog predictions as input (in OrthoXML or tab-delimited text format) and returns performance estimates on a variety of tests in comparison with other submissions in the form of summary graphs and downloadable raw results (Figure 1). Since its introduction, it has processed over 1000 user submissions. In the rest of this section, we describe improvements to the benchmark service, provide an update of the results since the last publication, and conclude with open problems in orthology benchmarking.

### Improving the generalized species tree discordance test

The most substantial change to the benchmarks themselves relate to the generalized species tree discordance benchmark. This benchmark assesses the quality of orthology predictions by comparing gene trees built from predicted orthologs with the underlying species phylogeny. The benchmark requires an undisputed, bifurcating species phylogeny. However, the set of species in the QfO proteome dataset includes some unresolved nodes—either due to inference uncertainty or genuine biological complications (such as incomplete lineage sorting, which causes bona fide local variation in the gene trees). When we introduced the benchmark (13), we showed how we can sample undisputed bifurcating species trees from the multifurcating QfO tree by randomly pruning the multifurcating nodes. In brief, we recursively traverse the multifurcating species tree and sample two random subtrees whenever a visited node has more than two subtrees. This results in a fully bifurcating subtree.

Since the benchmark was introduced, we noticed some biases in the resulting trees. Many gene trees covered only a few species, especially on the Last Universal Common Ancestor (LUCA) dataset. The reason for this skew is that, in addition to the uneven species sampling across the tree, the reference species tree is uneven in terms of resolved internal nodes—some parts are well resolved (i.e., contain binary splits only) while others are not, with some of the deep nodes placed under successive multifurcations. Due to the sampling procedure, such nodes were rarely sampled, leading to a bias in the species selection.

To reduce this bias, we improved the sampling of the species trees in the following way. We now select a fixed number of species (i.e. 10) uniformly, and check whether they form a bifurcating subset on our reference topology. If they do, we keep the sample, otherwise we repeat. This is repeated until we obtain 50,000 species tree samples. This fixed set of species trees is then used to sample gene trees according to the orthology predictions. The rest of the benchmark procedure remains unchanged: For each proposed species tree, we select for a random species a random gene and try to sample a path of orthologs along the species phylogeny. If we succeed in selecting a full path covering all species, we compute a multiple sequence alignment using MAFFT (16) for the set of protein sequences and compute a least squares distance tree. The topology of the gene tree is then compared to the expected species phylogeny. In the benchmark, we report the average topological distance of the gene tree to the species phylogeny using the Robinson-Foulds distance (17).

Because the distribution of trees generated by the new sampling strategy—implemented from the 2018 benchmark set onwards—differs from the previous strategy, the performance measures (recall in terms of successfully sampled trees and error in average Robinson-Foulds distance) are not directly comparable to the previous versions. However, the tradeoff each method makes between recall and error remains substantially unchanged.

### Moving to OpenEBench, the ELIXIR platform to support community-led scientific benchmark efforts

Despite the clear value of the benchmark service to the community, maintaining individual infrastructures for supporting community-led benchmark efforts is costly and labour-intensive. For this reason, ELIXIR—the pan-European infrastructure for life sciences—contributes to the development and maintenance of a platform to support community-led scientific benchmark activities (18). OpenEBench provides a platform where communities with different needs of support for their benchmark activities can benefit from a modular architecture. OpenEBench implements the FAIR data principles (19) and has adopted the use of software containers for an easy deployment of benchmark workflows. Indeed, the QfO benchmark service now uses software containers within Nextflow workflows (20). This implementation allows the QfO community to more easily add new and/or remove existing benchmarks to future challenges. The modular design also contributes towards the maintainability of the codebase as changes in one part of the workflows does not affect other parts.

**Figure 2.**
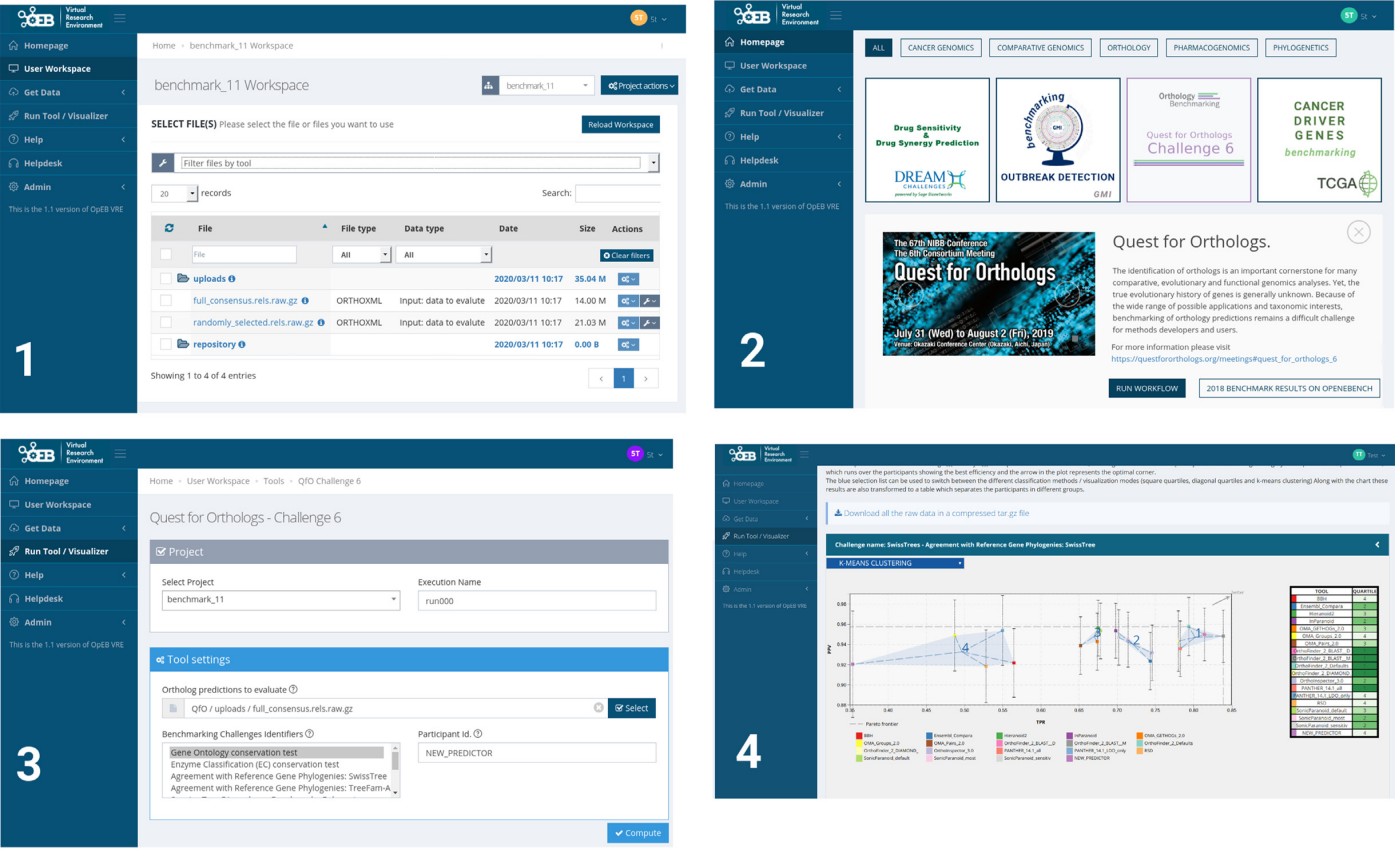
Functionality of the OpenEBench platform for ortholog benchmarking: 1) Upload the data to evaluate an OrthoXML file containing participant's orthologs predictions. 2) Select the benchmarking event among the available in the Virtual Research Environment. 3) Set the parameters for the benchmarking run. 4) Compare the results of the new predictor against the rest of the participants with the available visualizers.

For orthology method developers and other end-users, the move to the OpenEBench Virtual Research Environment (21,22) as a backend provides faster and more scalable computations, as well as more flexible output graphs implemented using modern web technologies (Figure 2). For instance, the choice of methods included in the graphs can be selected interactively. All benchmarking functionality is available as an anonymous user (no login required) or as a registered user. In addition, registered users can store their results on OpenEBench and, optionally, make them publicly available through the platform.

### Benchmark results on the 2018 proteome dataset

Because it can take up to a year for the main orthology resources to submit their predictions on a new proteome set, the latest results were obtained on the 2018 QfO Reference Proteomes set. The benchmark service provides public results for 10 different resources or algorithms, some of which have multiple variants (either because the resources can be run with different options, or because they produce multiple types of orthologs) resulting in a total of 19 different orthology predictions.

**Figure 3.**
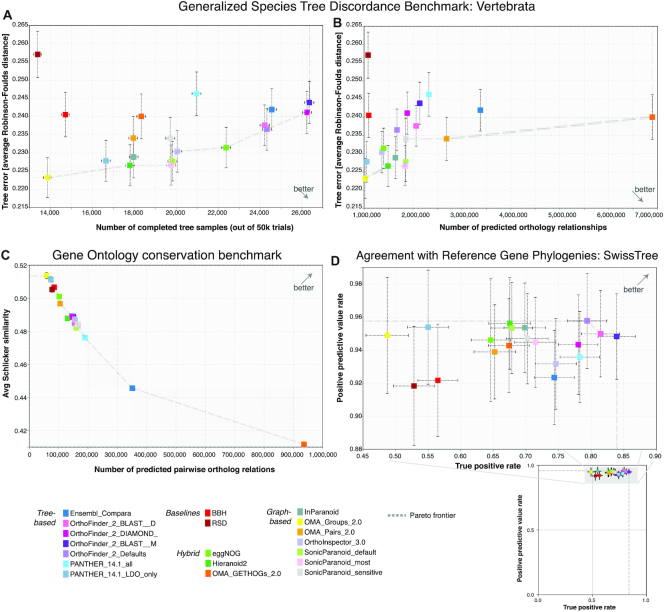
Excerpts from the public benchmark results on the 2018 QfO reference dataset. (**A, B**) The choice of recall measure (x-axis) can have a big impact on the generalized species tree discordance test. (**C**) In the Gene Ontology conservation benchmark, nearly all methods lie on the ‘Pareto frontier’ (dotted line). (**D**) In the reference gene tree benchmark based on SwissTree, most methods have a similar precision (y-axis) but vary considerably in recall (x-axis). Error bars indicate 95% confidence intervals. Note that the axis ranges have been chosen to optimise the separation of the data points. As such they do not show proportional changes in accuracy measures and so careful interpretation is required. The full results, with interactive viewing options (selection of methods included, choice of precision and recall measures, display of full axis range etc.), are accessible at https://orthology.benchmarkservice.org.

A top performing method is expected to lie on the ‘Pareto frontier’, i.e. the curve defined by data points which are not outperformed by any other ones in both precision and recall. For some of the benchmarks, many methods lie on or near the Pareto frontier, and thus mainly differ in terms of the tradeoff between precision and recall (Figure 3). This is because poorly performing methods and/or erroneous submissions are typically not released publicly by the submitter. The classical Bidirectional Best Hit (BBH) method (23) and its distance-based counterpart Reciprocal Shortest Distance (RSD) (24) tend however to perform worse than others, likely due to their inherent inability to deal with one-to-many or many-to-many orthologs (25).

In the previous benchmark, tree-based methods did not outperform graph-based methods, which came as a surprise because the former are typically more computationally intensive. This result still broadly holds, but the distinction is becoming less meaningful, due to the emergence of methods combining aspects of both tree- and graph-based methods—such as OrthoFinder 2 (26), Ensembl compara (27), Hieranoid (28), OMA GETHOGs (29) or eggNOG (30).

We also observe that recent changes in the algorithm of Ensembl compara (https://www.ensembl.org/info/genome/compara/homology_method.html) and OMA GETHOGs (29) result in a large increase in the number of reported orthologs (2.5 fold increase for OMA GETHOGs, 15.9% increase for Ensembl compara, both on the 2011 dataset), which is apparent in the 2018 dataset results (Figure 3B). This increase does however not translate into a larger number of completely sampled trees in the species tree discordance test (Figure 3A). The reason for this discrepancy between the two recall measures is not clear at this stage, and resolving it will require more investigation. The source of the very large number of orthologs appears to be deep many-to-many relationships, which may be hard to sample complete trees from. Meanwhile, the discrepancy illustrates the benefit of reporting more than one kind of recall measure in the benchmark.

## PUBLIC PREDICTIONS OF ORTHOLOGS USED BY ALLIANCE OF GENOME RESOURCES (AGR)

One way to take advantage of the different orthology inference algorithms is to combine them into a meta-prediction of orthology (8). Several meta-prediction resources have been developed, including HGNC Comparison of Orthology Predictions (HCOP) (31), Drosophila Integrative Ortholog Prediction Tool (DIOPT) (32), OrthoList (33), and Ortholog Scanner (ORCAN) (34). Because ortholog benchmarking has shown that the primary difference between orthology prediction methods lies in their precision versus recall tradeoff (13), agreement between different methods can serve as a proxy for prediction confidence level. HCOP and DIOPT report a simple count of the number of methods that agree on a given prediction.

**Figure 4.**
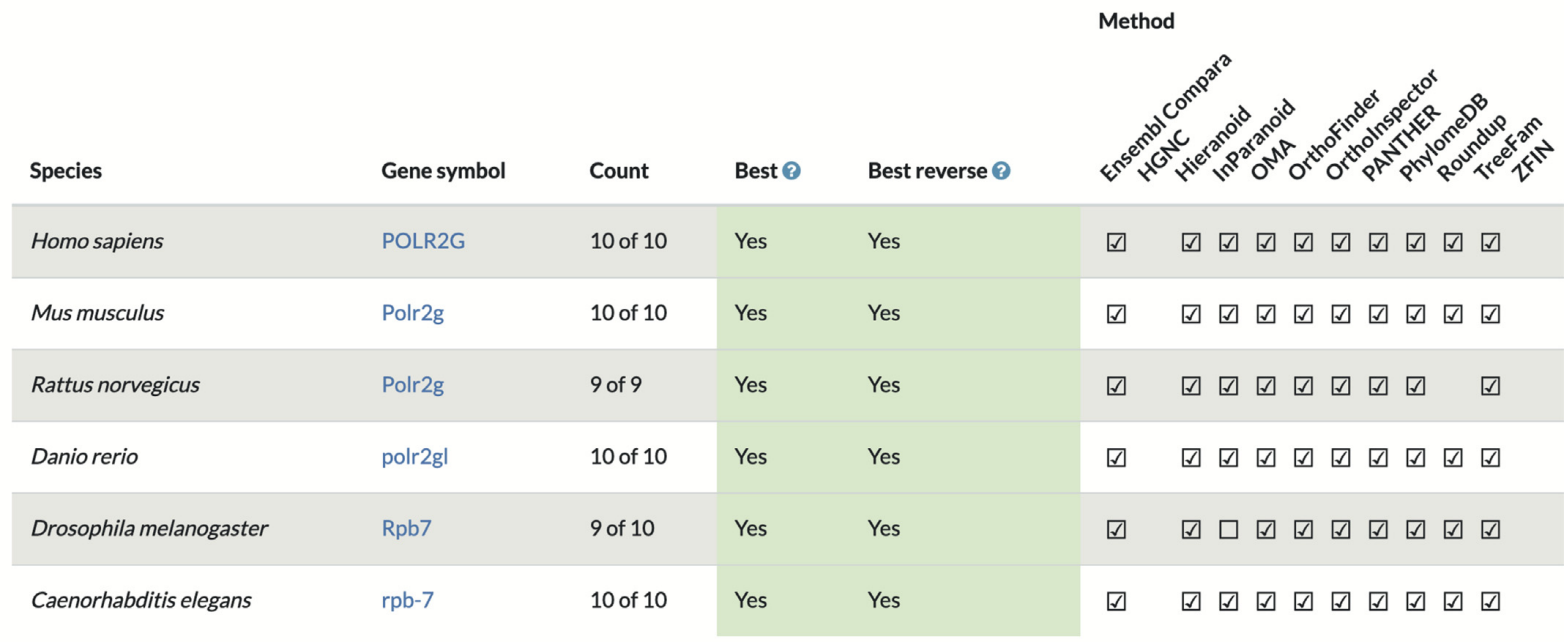
Consensus orthology call for the gene RPB7 in *S. cerevisiae*, available from https://alliancegenome.org.

Recently, a new comparative genomics resource, the Alliance of Genome Resources (Alliance) (14) has been established to enable analyses across data for the human genome and the major model species for studying human biology (‘model organisms’), mouse, rat, zebrafish, fruit fly, nematode worm and Baker's yeast. Orthology is central to relating the experimental findings in each model organism to human biology, as gene function is generally conserved over long evolutionary time periods (35,36). The Alliance integrates predictions from all of the top-performing methods, using the most recent assessment from the Orthology Benchmarking Service (Figure 4). Currently, the inclusion criterion is performing at the Pareto frontier of precision vs. recall, for at least one of the standard benchmarks; orthologs are integrated using DIOPT. Through manual review and testing, several different ortholog prediction stringency levels have been defined by the Alliance. The Alliance provides ortholog predictions relating seven different genomes (human plus the six model organisms above) on interactive web pages (https://alliancegenome.org) as well as via Application Programming Interfaces (APIs; https://www.alliancegenome.org/api/swagger-ui/, Homology API).

The standardization of different ortholog inference predictions on the same set of UniProt Reference Proteomes (37), and availability of these predictions in standard formats (https://ortholog.benchmarkservice.org) facilitates meta-prediction. Previously, different methods made predictions using different protein sets, with different identifiers and often even different protein sequences for the same protein-coding gene, requiring meta-predictions to implement complex mapping processes between these different identifier spaces and sequences. We encourage meta-prediction method developers to download the most recent predictions from the benchmarking service website, as methods are under constant development and improvement, and the protein-coding gene sets are also being iteratively improved at each release. In addition to Reference Proteomes pipeline improvements, all of the model organism resources, and the Human Gene Nomenclature Committee for human genes, work closely with the UniProt resource to ensure that the Reference Proteomes reflect any updates in gene structure annotations.

## OPEN CHALLENGES IN ORTHOLOGY BENCHMARKING

The benchmark server has been updated and improved in many aspects. Orthology benchmarking is, however, a fundamentally difficult problem. To directly gauge performance in terms of precision and recall, a truth set needs to be known, which is often elusive when it comes to gene phylogenies. Instead, benchmarking is performed indirectly, in different ways, each of which with their own advantages and drawbacks (5). Although the current server casts a wide net by including six benchmarks where each benchmark presents results in up to four variants, there remains a number of open challenges. To make sure that the benchmarks capture the algorithms’ performance in a fair and unequivocal way, and that an algorithm's ability to find more complex orthology assignments are premiered, should be the overarching goals. In addition, the benchmark should be resistant to gaming attempts.

One aspect that has been difficult to score fairly is the coverage or recall. Simply counting the number of uploaded orthologs is not optimal, because an algorithm that generates large amounts of lower quality orthologs may not be much penalized for this in terms of precision. This is especially true for the species tree discordance benchmarks, which only sample a small amount of the uploaded orthologs. Therefore, coverage is by default measured with ‘number of completed tree samplings’ in these benchmarks. A completed tree sampling means that ortholog pairs could be sampled that connect 10 randomly selected species in a daisy-chain fashion. While this is a clear improvement, certain issues remain such as the optimal number of species per tree, and whether species should be selected at equal frequency, or proportional to their proteome size, or their number or orthologs. For the EC and GO function-based benchmarks no sampling is done, hence only the number of uploaded orthologs is used as a proxy for recall.

The current benchmark server only considers ortholog pairs as input. The reason for this is that it is a common ground that all ortholog providers can support. On the other hand, many algorithms infer larger groups of orthologs from multiple species, and this structure is lost when ortholog pairs are extracted from the groups for benchmarking. It would therefore be desirable to also benchmark larger and more complex ortholog assignments. One possibility would be to follow the strategy of OrthoBench (38) which considers all descendants below a given species tree node (i.e. ancestral species) in different species to be orthologs. However, if a gene duplication is followed by speciation, which is common, outparalogs would be considered orthologs, which is wrong (39). Furthermore, there are numerous taxonomic levels to perform such an analysis on, and the results at each level will likely be different. A better approach is probably to assess submitted orthogroups directly. One could do this for instance by removing duplications by randomly picking one child at each duplication node and then test whether the resulting tree only contains speciations by examining if it corresponds to the species tree. Several issues would need to be resolved, however, such as the number of iterations of random duplication removals, whether scores should be weighted by orthogroup size, and whether sampling should be allowed for efficiency reasons.

Duplications after speciation events give rise to inparalogs (2), and from a functional point of view it is important to capture inparalogs, which are co-orthologs to genes in other species, as they may encode redundant functions. However, the current benchmark server based on ortholog pairs does not reward inparalogs, and the above proposed scheme of orthogroup benchmarking would also not, as inparalogs would be removed. In the current server, including inparalog predictions does not increase the recall, i.e. the number of successful species tree samplings, and also not the precision, which may actually decrease when including the less conserved co-orthologs. If prediction of inparalogs were to be rewarded, probably some rather explicit score such as ‘inparalog coverage’ would need to be measured, which sums up the number of inparalogs in submitted or sampled orthogroups.

All benchmarks on the server consider whole-protein orthology only. However, it has been shown that orthology analysis of individual protein domains can be beneficial, as orthologous domains may be missed on the whole-protein level (2,40). A few algorithms exist that can infer orthology on the domain level (e.g. 41–43). A drawback of the current benchmark server is that finding domain-level orthologs will not be rewarded since the benchmarks are not domain-aware, and therefore submitting bona fide domain-level orthologs will reduce the score. A potential solution would be an additional benchmark in which the QfO Reference Proteomes sequences are split into domains, e.g. by using Pfam (44), and then operate on these subsequences. This would only work for the species tree discordance benchmark, as the other benchmarks use either reference trees or functional annotations from GO or EC that are only available on the whole-protein level.

OpenEBench provides an opportunity to improve existing benchmarks and include new ones as a better understanding of existing results emerges. The use of a modular architecture based on software containers opens the possibility to quickly incorporate new benchmarks into the existing collection and apply them to the existing collection of submitted datasets. Moreover, the alignment of OpenEBench with the standards promoted by different communities will contribute to the adoption of sustainable solutions over time. This transition will benefit the QfO community by allowing it to focus on refining existing benchmarks and developing new ones rather than investing in maintaining an individual infrastructure.
